# The inclusion of disability and aging in COVID-19 hygiene behavior change interventions across low-and middle-income countries: A review using the COVID-19 Inclusive WASH Checklist

**DOI:** 10.3389/fpubh.2022.1024850

**Published:** 2022-11-21

**Authors:** Jane Wilbur, Sharika Ferdous, Lorraine Wapling

**Affiliations:** ^1^International Centre for Evidence in Disability, London School of Hygiene and Tropical Medicine, London, United Kingdom; ^2^Infectious Diseases Division, International Centre for Diarrhoeal Disease Research (ICDDR), Dhaka, Bangladesh; ^3^Department of Epidemiology and Public Health, University College London, London, United Kingdom

**Keywords:** COVID-19, disability, aging, caregivers, hygiene, water and sanitation

## Abstract

**Introduction:**

People with disabilities and older adults face a high risk of dying from COVID-19. Handwashing with soap and sanitizing surfaces were recommended to disrupt COVID-19 transmission. Yet, in many low-and middle-income countries (LMICs), these populations have inadequate access to water, sanitation and hygiene (WASH) and are not reached by public health campaigns. The Hygiene Behavior Change Coalition (HBCC) was set up to limit the spread of COVID-19 in LMICs. Twenty organizations working across 37 countries were funded to encourage populations to adopt recommended personal hygiene behaviors. This study aims to review the inclusion of disability, aging, and caregiving in HBCC grantee interventions.

**Methods:**

A COVID-19 Inclusive WASH Checklist, which incorporates core concepts of human rights, was developed to support the inclusion of disability, aging and caregivers in interventions. The Checklist was applied to 137 documents submitted to donors within the HBCC fund to assess inclusion. Eligible grantee programme documents related to HBCC-funded projects were identified between August 2020 and January 2021. Feedback was provided to grantees recommending how to strengthen the inclusion of disability, aging, and caregiving.

**Results:**

Most organizations identified people with disabilities, older adults and caregivers as target groups, but targeted activities to include them were scarce. Where efforts were made, immediate needs rather than rights were addressed. For example, the construction of accessible handwashing facilities featured more prominently than ensuring the participation of these groups. Examples of the coverage of core concepts in interventions included generating data with these groups and developing interventions accordingly. Limitations to inclusion were inconsistent organizational approaches, inability to monitor media campaigns, and inadequate coverage of disability and aging in donor's grant funding mechanisms.

**Conclusion:**

To ensure these populations benefit from efforts, they must be explicitly identified as target groups, with assigned actions that are monitored; efforts must go beyond accessible WASH services to ensure the meaningful participation of these groups. The COVID-19 Inclusive WASH Checklist supports this but requires further testing to assess its appropriateness and effectiveness.

## Introduction

Fifteen per cent of the global population has a disability, meaning a “long-term physical, mental, intellectual or sensory impairments which, in interaction with various barriers, may hinder their full and effective participation in society on an equal basis with others” (1, 2). In 2020, an estimated 727 million people were aged 65 years and over globally (3) (“older adults”), of whom approximately a third have a disability (1). An estimated 190 million people rely on informal and professional caregivers for assistance (4). Moreover, many people providing care to others during the COVID-19 pandemic are older adults (5–9).

People with disabilities, older adults, and older adults with disabilities are at a higher risk of COVID-19 (10–12). Seventeen per cent of the United Kingdom population has a disability but constituted 60% of COVID-19 deaths, and over 90% of deaths are among people aged 60 years or older (13). A recent scoping review of 58 articles, mainly from high-income countries, found COVID-19 infection rates were higher for people with disabilities living in residential care settings, which authors attribute to the crowded environment (12). These excess risks have important implications for COVID-19 control strategies as people with disabilities, older adults, and older adults with disabilities make up a large proportion of the global population.

During the COVID-19 pandemic, social distancing, wearing face masks and personal hygiene measures, such as handwashing with soap and water and keeping surfaces clean, were recommended to disrupt COVID-19 transmission routes (14–16). These activities can present challenges for people with disabilities, older adults, and older adults with disabilities: social distancing is challenging for people who rely on caregivers; face masks inhibit lip reading; and many are unable to avoid touching contaminated surfaces, such as assistive technologies and products. Furthermore, public health campaigns are often not inclusive or accessible (17), and these groups often face barriers to attending healthcare services (e.g., inaccessible transport and health facilities, financial constraints, and negative attitudes) (17, 18). These issues may be further exacerbated in resource-constrained low-and middle-income countries (LMICs) (17, 18).

More specifically, people with disabilities, older adults, and older adults with disabilities face a range of barriers to accessing and using water, sanitation, and hygiene (WASH) facilities and services, which are vital for disrupting COVID-19 transmission (15, 16, 19–22). These barriers intensify during emergencies (23, 24). These include inaccessible water points, handwashing and bathing infrastructure (19, 21, 22, 25, 26), limited water quantity (7) and the ability to afford soap (5, 6, 18, 27, 28). Additionally, caregivers might not prioritize the WASH needs of the individual they support or may not be informed about how to do this effectively (26, 29, 30). Caregivers may also face difficulties transferring information about COVID-19 preventative measures to individuals they support (31, 32).

In accordance with basic human rights principles, people with disabilities, older adults, and older adults with disabilities must be included in all COVID-19 responses (2, 33–35). For instance, the United Nations (UN) recognizes that the human rights to water and sanitation are essential for realizing all other human rights. The UN Convention on the Rights of Persons with Disabilities (CRPD) includes Article 11, stipulating that all humanitarian responses should include people with disabilities (2, 36). Yet a review of 23 global guidance documents on WASH and COVID-19 revealed that only one-third referenced people with disabilities (aging was not part of the study) (37).

In early 2020, Unilever and the Foreign, Commonwealth & Development Office (FCDO) launched the £100 million Hygiene Behavior Change Coalition (HBCC) to mount a rapid response to contain and limit the spread of COVID-19 in LMICs. The HBCC aims to reach up to a billion people, raising awareness and changing behavior, to ensure people wash their hands regularly with soap and disinfect surfaces. Twenty-one organizations were funded to deliver interventions in 37 countries across Sub-Saharan Africa, South Asia, East Asia and Pacific, Latin America, Middle East and North Africa regions. Projects ran from April 2020 to June 2021; activities included the distribution of hygiene products, such as hand soap and sanitisers, installing handwashing facilities at public locations, holding handwashing demonstrations, and running social, digital and mass media campaigns that promoted handwashing with soap, maintaining social distancing, respiratory hygiene, and surface cleaning. At the request of FCDO, the International Center for Evidence in Disability (ICED) at the London School of Hygiene & Tropical Medicine delivered a seminar on the importance of including disability and aging in HBCC-funded projects in April 2020.

## Methods and materials

### Study aims and objectives

This study aims to explore the inclusion of disability, aging, and caregiving within HBCC grantees' efforts to prevent COVID-19 transmission in low- and middle-income countries (LMICs).

Specific study objectives were to: (1) develop a COVID-19 Inclusive WASH Checklist to support the inclusion of people with disabilities, older adults (aged < 60 years), and their caregivers in hygiene behavior change projects to prevent COVID-19 transmission, (2) apply the checklist to HBCC grantees' programme documentation to understand the number of organizations that targeted people with disabilities, older adults, and caregivers in their interventions, (3) explore the core concepts of human rights considered within these organization's approaches and score the quality of these commitments, and (4) identify examples of the coverage of core concepts in interventions, and limitations faced in achieving inclusion.

In this article, we present the development of the COVID-19 Inclusive WASH Checklist and how it was applied to review HBCC grantees' programme documents (objectives 1 and 2) before documenting the results of that review (Objectives 3 and 4).

### Developing the COVID-19 Inclusive WASH Checklist

A COVID-19 Inclusive WASH Checklist, which incorporates human-rights principles, was developed and finalized after review by WASH and disability specialists at the LSHTM.

The purpose of the COVID-19 Inclusive WASH Checklist was: 1) to enable the systematic analyses of included materials of inclusion of people with disabilities, older adults, and caregivers and 2) to provide practical guidance for WASH practitioners wishing to ensure people with disabilities and older adults benefit from COVID-19 responses (38). The checklist can be applied during the planning, design, monitoring and evaluation of projects, and recommendations can be made to enhance inclusion throughout the project cycle.

We define inclusive WASH as ‘a process which addresses the barriers to accessing and using WASH services faced by people vulnerable to exclusion, including people with disabilities, older adults, people living with chronic illness, women, girls, transgender and non-binary people' (30).

#### Search strategy

The search strategy was developed to identify peer-reviewed studies and gray literature documents relevant to WASH, disability, and COVID-19. The review included all countries but was limited to papers published after 2010 written in English. Searches were conducted in July and August 2020 across two online databases: Global Health and PubMed through Ovid SP, and gray literature was gathered through Google Scholar, Google, and Twitter and discussions with WASH and disability specialists. Search terms were created to capture four main concepts: WASH, disability, aging, and COVID-19.

#### Inclusion/exclusion criteria

Eligible papers included technical briefs, guidance notes, frameworks, toolkits, blogs, primary research, research reports, conference papers, and commentaries. No exclusion criteria were set for the world region. Papers published before 2010 [when the UN recognized the human right to safe drinking water (36)] and non-English documents were excluded. Papers were required to consider WASH service provision during humanitarian emergencies, including COVID-19, for people with or without disabilities and/or older adults, and COVID-19 responses for people with disabilities and/or older adults.

#### Study selection and description of papers

Papers identified through the search were exported to EndNote version X9, duplicates were removed, and papers were screened against the eligibility criteria. Fifty-one papers were identified (peer-reviewed *n* = 3; gray literature *n* = 48). The authors then shared these papers with WASH practitioners. They asked them to identify those they had used and which were most helpful in guiding the inclusion of disability and/or aging in their practice, thus narrowing down the focus from general WASH papers to those supporting the inclusion of disability and aging. The following five were identified through the two stage-screening processes.

1. *The EquiFrame framework*, adapted for WASH and disability, assesses the extent to which 21 core concepts of human rights are included in WASH policies and guidance documents (39). Each “core concept” of human rights has key questions and key language to support consistent analyses and scoring of policy content (39). The EquiFrame framework includes a quality of commitment score of 1 to 4: 1 = concept only mentioned; 2 = concept mentioned and explained; 3 = specific policy actions identified to address the concept; 4 = intention to monitor concept was expressed.
2. *The normative criteria to specify the right to water and sanitation* (40, 41). These criteria include non-discrimination, participation, availability, quality, acceptability, accessibility, and affordability.
3. *Key actions for disability-inclusive WASH and COVID-19* (42). This checklist includes 14 steps to ensure disability is factored into WASH and COVID-19 responses. They include involving people with disabilities and their representative organizations, addressing gender and disability issues and advocating for inclusive WASH responses to COVID-19.
4. *Key tool: Equity, non-discrimination and inclusion in WASH checklist* (43). This checklist covers 15 activities identified to ensure everyone benefits from WASH interventions. Actions range from conducting a situational analysis to providing subsidies for people unable to pay for water and sanitation services.
5. *Steps to ensuring people with disabilities, older adults, older adults with disabilities and their caregivers are included in all COVID-19 hygiene promotion programmes* (44). Fourteen recommended actions are to be applied throughout the total programme cycle. This cover conducting rapid reviews of the WASH-related barriers and challenges experienced by these groups, providing advice on how to keep support structures and assistive products clean and being evidence-driven.

### Development of the checklist's guiding principles and suggested activities

The EquiFrame, adapted for WASH and disability and which identifies core concepts of human rights, was used as the basis of the COVID-19 Inclusive WASH Checklist. However, as the adapted EquiFrame does not specifically relate to COVID-19 or aging, the content was mapped across the remaining four documents to make it relevant for COVID-19, WASH, disability and aging. Consequently, the EquiFrame 21 core concepts of human rights were reduced to 15. Core concepts were omitted if the content was absent within the above resources (numbers 2–5). Those excluded were *Liberty, Autonomy, Privacy, Contribution, Cultural responsiveness*, and *Prevention*.

As the lead author is an academic focusing on disability and WASH, with practical experience in designing, delivering, and evaluating inclusive WASH interventions, she developed “guiding principles” to meet the core concepts and “suggested activities” to support their achievement. A color-coded grade is included in our checklist based on the EquiFrame framework quality of commitment scoring criteria. An additional score of zero (the core concept was not mentioned) is included (see above). The Checklist was reviewed by co-authors, academics at the LSHTM working in WASH, and disability and WASH practitioners. Any suggested revisions were discussed and incorporated into the Checklist.

Supplementary material contains the COVID-19 Inclusive WASH Checklist. The checklist is presented twice, firstly in relation to disability and secondly to aging. Attention to caregivers is integrated into “suggested activities” under the disability and aging components, but the Family resource and Family support core concepts focus on caregivers.

Table 1 includes an example of *Non-discrimination* from the COVID-19 Inclusive WASH Checklist. Table 2 provides examples of project activities and how they would be scored against the core concept, *Non-discrimination*.

**Table 1:** Example of core-concept non-discrimination, against disability, from the COVID-19 Inclusive WASH Checklist.

**Disability**								
**Core concept**	**Guiding principles**	**Activity #**	**Suggested activities**	**0** = **Core concept not mentioned**	**1** = **Core concept only mentioned**	**2** = **Core concept mentioned and explained**	**3** = **Specific programme target and actions identified to address the core concept**	**4** = **Actions and targets monitored and evaluated, with results presented against core concept**
		1.1	Persons with disabilities are considered an 'at risk' group					
		1.2	Persons with disabilities included as a target population					
		1.3	Have a separate budget line for every activities related to disability, including staff capacity development, outreach for people unable to leave their home or who are self-isolating, and all other related programme and policy activities					
Non-discrimination	Intervention support the rights of people with disabilities with equal opportunity in accessing WASH services	1.4	Identify ways of engaging people with all types of impairments at all stages of COVID-19 programmes, from planning to evaluation					
		1.5	Ensure all impairment groups and genders are represented in Organizations of Persons with Disabilities					
		1.6	Work with community leaders, Organizations of Persons with Disabilities and disability service providers to identify households that include a person with a disability					
		1.7	Actively seek to include people with different impairments, ages, genders, and their caregivers					

**Table 2:** Examples of project activities and how they would be scored against non-discrimination for disability.

**Score**	**Non-discrimination core concept example**	**Corresponding activity in Table 1**
0 = Concept was not mentioned	No content	N/A
1 = Core concept only mentioned	We will target vulnerable populations (e.g., people with disabilities)	1.2
2 = Concept was mentioned and explained	Of the 100,000 people targeted, approximately 15,000 people experience some form of disability.	1.2
3 = Target and actions identified to address the core concept	Work with Organizations of Persons with Disabilities to identify up to 15,000 people with disabilities.	1.2, 1.6
4 = Actions and targets monitored and evaluated against core concept	In collaboration with Organizations of Persons with Disabilities, we identified 15,000 people with a disability	1.2, 1.6

### Identification of HBCC grantee documents for review using the COVID-19 Inclusive WASH Checklist

A search strategy was defined to identify relevant documentation submitted to Unilever (who then shared them with the FCDO) by the HBCC grantees. The search was conducted between August 2020 and January 2021. All documents submitted by grantees to donors were made available to the researchers.

Eligible programme documents for review had to relate to the HBCC-funded programmes explicitly. Included materials were funding proposals, project overviews, work plans, Theories of Change, progress reports, media and communication content, results frameworks, and budgets. Documents were excluded if they were published before the HBCC funding call or if they did not relate directly to the HBCC-funded projects. Organizational policies, such as counting user protocols, safeguarding and child protection, and recruitment, were excluded, as were the Curriculum Vitae of project staff. Table 3 presents the included materials.

**Table 3:** Included materials.

**Document type**	**N=**	**%**
Proposal and project overview	37	27
Workplan	11	8
Results framework	11	8
Budget	5	4
Media and communication content	16	12
Monitoring report	57	42
Total	137	100

### Applying the COVID-19 Inclusive WASH Checklist: Data extraction and analysis

We pilot-tested the COVID-19 Inclusive Checklist on two documents and revised it to address identified limitations. The revised Checklist was then applied to 137 eligible documents related to HBCC-funded projects. To ensure reliability and validity, two authors independently reviewed the content of included materials to identify the coverage of core concepts and then scored each reference. Any discrepancies were discussed before agreeing on final scores.

Each reference to a core concept that scored 3 or 4 (*Specific programme target and actions identified to address the concept*, and *Actions and targets monitored and evaluated, with results presented* respectively) were considered “high quality.” The following excerpt from a programme document is an example of the core concept, *Access*, because it provides sign language interpretation on television and therefore provides hygiene information in accessible formats for people with disabilities. As it includes specific actions (sign language interpretation), it is awarded a quality of commitment score of 3.

“*Mass media (TV, Radio & SMS) – [organisation's name] aired on Citizen TV to tap into its high reach and affinity during the reporting period, under the social inclusion agenda, we supported sign TV with content co-produced with the station to reach people living with disabilities, namely deaf persons.”* (*Access*, scored 3, referencing disability).

For each included document, we captured the number of times each core concept was mentioned, the total score across these and the average score. We then captured the number of references made to each of the 15 core concepts and the average score awarded across all documents. These are presented in this article.

### Ethics statement

As the inclusion of disability and aging was not part of the donors' funding criterion, HBCC grantees may not have factored this into their original aims and objectives. To acknowledge this, our feedback and advice to grantees about improving the inclusion of disability and aging in their projects was confidential and was not shared with donors. Grantees could decide if they had the resources to implement the recommendations or not, and this was not monitored by the authors of this study or the donors. To maintain confidentiality, grantees' names are omitted in this article.

## Results

### Coverage of core concepts against disability and aging and their quality of commitment scores

People with disabilities were explicitly identified as target groups within 18 organizations (90%), compared to 17 (85%) for older adults and 16 (80%) for caregivers of people with disabilities or older adults. Table 4 presents the frequency of each core concept referenced across all 137 documents (total references %) and the score awarded to each (average score).

**Table 4:** Frequency of references to core concepts and average scores across all documents, and the comparison between disability and aging.

**Core concept**	**Disability (*****n*** = **375 References)**			**Aging (*****n*** = **216 References)**			**Proportion of total references to disability and aging (*****n*** = **591)**	
	**Total references (n=)**	**Total references (%)**	**Average score**	**Total references (n=)**	**Total references (%)**	**Average score**	**Disability total (%)**	**Aging total (%)**
Non-discrimination	88	23%	1.4	61	28%	1.3	15%	10%
Individualized services	61	16%	1.8	34	16%	1.5	10%	6%
Entitlement/affordability	2	1%	1.0	2	1%	1.0	0%	0%
Capability based services	9	2%	2.0	1	0%	1.0	2%	0%
Participation	12	3%	1.3	3	1%	1.0	2%	1%
Coordination of services	7	2%	1.4	1	0%	1.0	1%	0%
Protection from harm	15	4%	1.8	4	2%	1.5	3%	1%
Integration	6	2%	2.0	0	0%	0.0	1%	0%
Family resource	31	8%	1.6	31	14%	1.5	5%	5%
Family support	0	0%	0.0	1	0%	3.0	0%	0%
Accountability	0	0%	0.0	0	0%	0.0	0%	0%
Capacity development	14	4%	1.5	9	4%	1.3	2%	2%
Access	74	20%	1.7	38	18%	1.3	13%	6%
Quality	50	13%	2.3	31	14%	1.7	8%	5%
Efficiency	6	2%	1.5	0	0%	0.0	1%	0%
Total	375	100%		216	100%		63%	37%

Table 4 shows that of the 591 references to core concepts across all documents, 375 (63%) were made to disability and 216 (37%) to aging. Across disability and aging, most attention was given to *Non-discrimination, Individualized services, Access, Quality, and Family resource*. The most neglected core concepts across disability and aging were *Entitlement/affordability, Family support*, and *Accountability*. However, between these, several core concepts were referenced minimally across all documents, especially to aging (e.g., *Capability based services, Coordination of services, Integration*, and *Family resource*, which received one reference against aging*)*.

**Figure 1:**
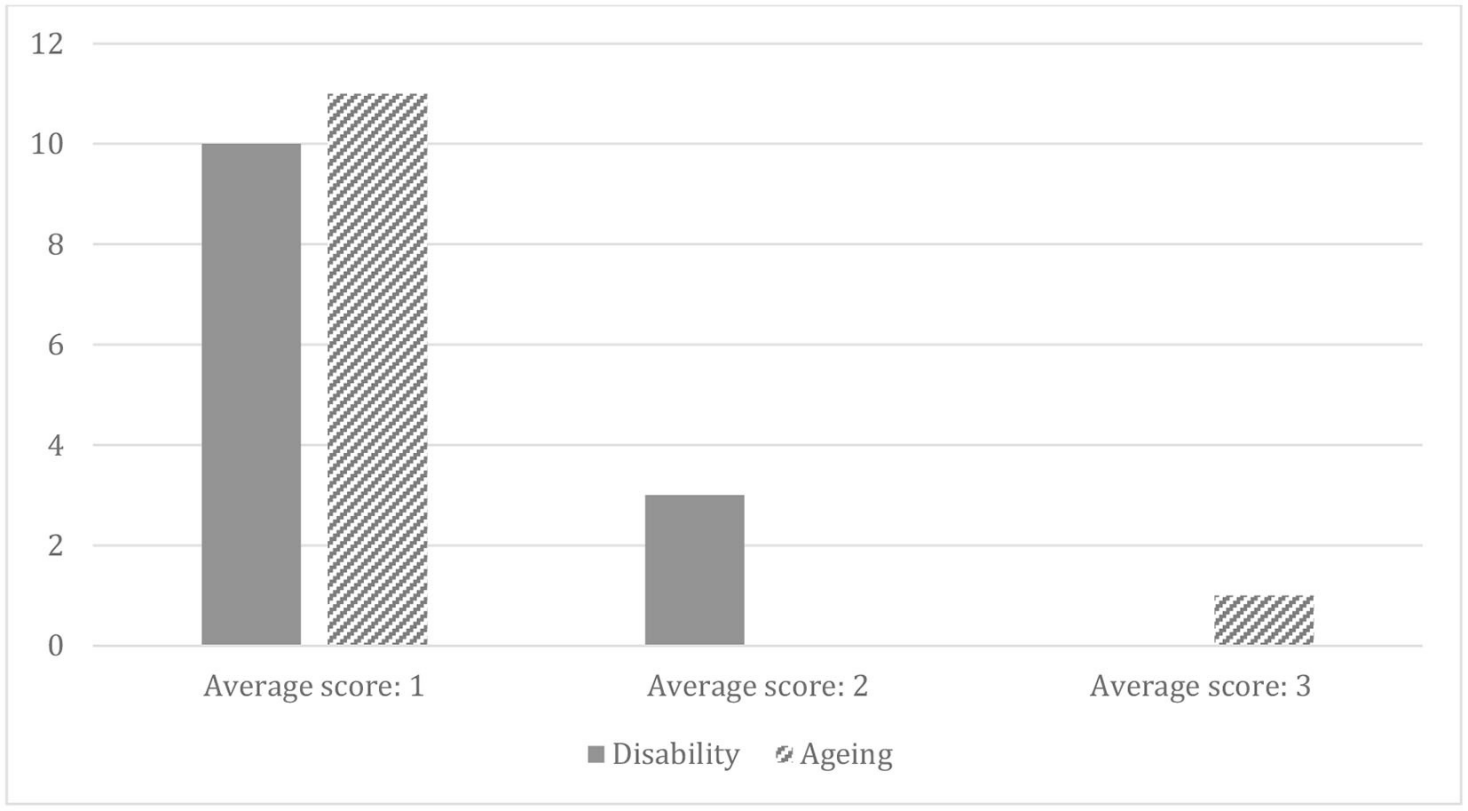
The average score awarded to references to core concepts across disability and aging.

All core concepts, bar *Family support* (aging), were given a low-quality average score of 1 or 2, meaning that, on average, they were not assigned specific activities or monitoring mechanisms. *Family resource* and *Family support* specifically focus on the inclusion of caregivers. Though the former was referenced frequently against disability and aging, the average score was low quality (Disability 1.6, Aging 1.5). *Family support* was the only concept to receive a high-quality score (aging 3.0), but this is from one reference across all documents, so it is not a strong indication of the quality of commitment. Figure 1 presents the average score awarded to references to core concepts in relation to disability and aging.

### Examples of how organizations included disability, aging, and caregiving in their hygiene behavior change interventions

Several examples of how organizations included disability, aging, and caregiving in their interventions were identified during the review process. In Syria, the following quote is taken from an organization's programme document, which shows that they target caregivers with their hygiene behavior change messages whilst also recognizing that caregivers are often older adults. Therefore, this relates to the *Family resource* core concept.

“*In overcrowded, multi-family, multi-generational households, older people frequently provide childcare while parents go out to work (particularly as schools are currently closed). This makes shielding or isolation impossible but offers an additional audience for messages focused on carers.”* (Family resource, aging).

Within the same project, community volunteers were also trained to adapt hygiene behavior change activities to suit the needs of people with different impairments. This is an example of *Capacity building* in relation to disability as frontline staff were trained to understand disability-inclusive WASH.

In Fiji, an organization collaborated with the Pacific Disability Forum during project design to ensure hygiene behavior change messages were accessible to people with visual and hearing impairments. This is an example of *Capability-based services* as the organization partnered with Organizations of Persons with Disabilities to ensure the programme's activities include people with disabilities.

*Individualized services* were referenced frequently in project documents across disability and aging. In Sierra Leone, one organization provided hygiene and menstrual hygiene kits to households that included people with disabilities *(Individualized services* and *Access)*. Safety issues related to using handwashing facilities in public settings were also considered *(Protection against harm)*. The following excerpt from a programme document shows that the organization recognized that disability and gender intersect to increase marginalization and designed activities to mitigate that.

“*Hygiene and menstrual hygiene kits will supply soap, buckets with taps, water storage containers and sanitisers for the most vulnerable households, including female carers and people with disabilities, to promote frequent handwashing and hygiene practices.”* (Individualized services, disability).

In Sri Lanka, Zambia and Nigeria, organizations constructed handwashing facilities accessible for people with mobility impairments, thus showing consideration for aging and disability. This activity contributes to achieving the guiding principle for the *Individualized services* core concept: “Intervention supports the rights of people with disabilities/older adults with individually tailored WASH services to meet their needs, choices and impairment so that they can carry out COVID-19 protective measures.”

In Myanmar, an organization conducted a rapid assessment to gather information about the experiences and requirements of older adults and people with disabilities *(Quality)*. Data were collected across demographics, knowledge, practices, and information needs. These were used to develop a hygiene behavior change campaign and identify key messages and dissemination platforms. The organization also used the Washington Group Short Set of questions to classify disability and over 60 years for aging (45) *(Quality)*.

### Limitations faced in achieving inclusion

A key limitation to including people with disabilities, older adults, and caregivers in HBCC interventions was that there was no specific encouragement from donors to be inclusive apart from the seminars delivered to HBCC grantees and donors through this review. FCDO state that their funding should be disability-inclusive (46), but proposals and reporting formats did not explicitly encourage or require the inclusion of disability and aging within interventions. For instance, disaggregated data was requested for gender, refugee / internally displaced person status, and rural and urban location, but not for disability or aging. Through this study and the broader evaluation of the HBCC funding [documented separately (47)], the donor reporting templates were revised in September 2020 to ensure organizations could document the inclusion of people with disabilities, older adults and caregivers in their projects, as well as disaggregate data across these groups. This change led to a greater breadth of data provided.

Few organizations systematically applied access and inclusion across all projects in the funding portfolio, meaning the inclusion of disability and aging was not mainstreamed across the whole organization. For instance, some organizations carrying out similar interventions in multiple countries reported the production of Braille/large print materials in only one or two locations or the construction of physically accessible facilities in a small number of places. Furthermore, few organizations included people with disabilities and older adults, or organizations representing these groups, in planning or monitoring activities *(Participation* and *Capability-based services)*.

Efforts to monitor the effects of mass media campaigns on the hygiene behaviors of people with disabilities, older adults or caregivers were absent *(Quality)*, but organizations requested support to do this. In monitoring reports, assumptions were made that generalized messaging impacted everyone who accessed them. However, without targeted market research, this assumption could not be verified.

## Discussion

Our study aimed to explore the inclusion of disability, aging, and caregiving within HBCC grantees' efforts to prevent COVID-19 transmission in LMICs. We noted several key results that will be discussed in relation to existing literature to demonstrate how this study contributes to current discourse.

Most HBCC grantees identified people with disabilities and older adults as target groups. This demonstrates that stakeholders are aware of the importance of ensuring COVID-19 WASH interventions are inclusive. However, the quality of commitment scores across all groups was low. Three other studies explored the inclusion of disability in WASH-related policies and guidance documents from Nepal (48), Cambodia and Bangladesh (39), and globally within the COVID-19 pandemic (37) by applying similar methods to those used in our study. Across all these studies, our review notes the most significant number of low-quality average scores. This indicates that these populations, who face a high risk of dying from COVID-19, may not have benefited equally from the HBCC-funded interventions.

A consistent finding across studies which explore inclusive WASH is that caregivers are not recognized for the critical support roles they often play in supporting people with disabilities and older adults with WASH and maintaining personal hygiene (21, 26, 30, 49–51). Our study found a greater emphasis on caregivers *(Family resource)* than in other studies (37, 39, 48). As noted above, though this indicates an awareness of caregivers' importance, this attention did not translate into clearly articulated actions for this group, meaning they were also unlikely to benefit extensively from these efforts.

These findings indicate that even though HBCC grantees understand the importance of inclusion, many struggled to mainstream it across their portfolio of funded projects. This chimes with a recent gap analysis that aimed to inform inclusive humanitarian responses; it noted that the operationalisation of inclusion is difficult to achieve, especially at the start of a humanitarian crisis, even though guidance exists (52).

We found that the attention to specific core concepts across disability and aging was similar. However, the total number of references made to individual core concepts was limited, particularly about aging, which was woefully low. Across this and other similar studies, *Individualized services* and *Access* are referenced consistently highly, whilst *Participation, Capability based services*, and *Accountability* receive little attention (37, 39, 48). Some may think this unsurprising because many governments, organizations, and institutions aim to increase access to WASH services, particularly in an emergency setting. Yet, for WASH services to be appropriate, acceptable, and sustainable, they must ensure the meaningful participation of target groups in the design, delivery, monitoring, and evaluation. People with disabilities, older adults, and organizations representing these groups must hold leadership roles with decision-making responsibilities. By tackling social exclusion, marginalization, and structural inequalities and improving access to WASH services, organizations can support people with disabilities, and older adults to increase their confidence, have greater control over resources, and better equip them to demand their rights to water and sanitation. This is in line with “Building Back Better,” which is the humanitarian strategy aimed at increasing people's resilience to future disasters (Priority Area 3 and 4) (53, 54).

In our results, we highlighted examples where HBCC grantees were incorporating disability, aging, and caregivers in their programmes, such as gathering data from target groups, analyzing it to understand requirements and designing appropriate interventions, and applying an intersectional lens in their analysis and intervention design. In the second phase of this study, a mixed-methods evaluation will be conducted to explore the inclusion of people with disabilities, older adults, and caregivers in HBCC-funded projects in Kenya, Indonesia, Zambia, Sierra Leone, and Bangladesh. Examples of promising inclusive-WASH practices will be documented in greater depth so that other development and humanitarian actors can integrate them into their interventions (47).

We also noted consistent challenges faced in mainstreaming inclusion in HBCC interventions. Of note was that funders' documentation templates did not encourage the explicit inclusion of people with disabilities and older adults. After the second quarter reporting formats were adjusted to incorporate disability and aging systematically, project documentation gained higher quality commitment scores. This suggests that reporting formats make a difference in sign-posting organizations to consider designing interventions with specific populations in mind. The FCDO has a Strategy for Disability Inclusive Development, which includes minimum standards for disability inclusion across all of its work (46). Additionally, humanitarian sector guidelines emphasize the importance of including people with disabilities and older adults in interventions, such as the Sphere Handbook (53) and Core Humanitarian Standards (55). Therefore, all donors funding humanitarian efforts should incorporate these.

Since completing this review, the FCDO and Unilever released a second HBCC funding stream in which the inclusion of disability and aging in interventions is a key criterion. They have also stipulated that all grantees must use the COVID-19 Inclusive WASH Checklist in their programme cycle, from design to evaluation.

Finally, organizations found monitoring and evaluating mass media hygiene behavior change campaigns difficult. Much can be learnt from existing literature, including the Behavior Centered Design (56, 57) and evaluations of mass media campaigns of anti-drug, alcohol and smoking campaigns (58–60). These studies gathered process monitoring data to understand the extent to which the campaign was delivered as intended and exposure of the target groups to the campaign and conducted outcome and impact evaluations. HBCC grantees could use such methods to assess their hygiene behavior change campaigns.

### Implications for future research and practice

To systematize inclusion within WASH interventions, all donors should sign-post the inclusion of people with disabilities, older adults, older adults with disabilities and caregivers in all documentation, from calls for proposals to monitoring, reporting and evaluation. They should also include disability and aging within the funding selection criterion. This would encourage organizations to explicitly consider inclusion within their WASH programmes, including COVID-19 WASH responses.

Though the COVID-19 Inclusive WASH Checklist provides practical guidance on incorporating disability, aging, and caregiving in interventions, further research in different settings is required to assess if the checklist effectively supports inclusion during COVID-19 and future humanitarian responses.

### Review strengths and limitations

A key strength of this study is that we developed and applied a structured tool, the COVID-19 Inclusive WASH Checklist, to analyse document content. To ensure transparency, we assessed data independently and compared results before finalization. We supported organizations to improve inclusion during the intervention by providing individualized feedback and recommendations. Another strength is that the donors encouraged and facilitated reflection and learning to enhance the inclusion of people with disabilities and older adults in COVID-19 WASH responses.

Some limitations must be considered when interpreting the study results. For instance, organizations might not have fully documented their inclusive approaches due to a lack of sensitivity to inclusion issues in standard reporting mechanisms. We did not interview any HBCC grantees separately to gather additional information. This will be addressed in the mixed-methods evaluation that will explore the inclusion of disability, aging, and caregiving in HBCC-funded projects in a greater-depth (47).

## Conclusion

This study demonstrates that the inclusion of disability, aging, and caregiving within HBCC grantees' efforts to prevent COVID-19 transmission in LMICs was not consistently achieved. To ensure these populations benefit from efforts, people with disabilities, older adults, and caregivers must be explicitly identified as target groups. Specific programme targets and actions must be determined to address a wide range of core concepts; progress toward achieving these must be monitored, evaluated, and reported on. While ensuring that WASH services can be accessed and used by these groups, ensuring their meaningful participation in all stages of the programme cycle is equally essential. The COVID-19 Inclusive WASH Checklist provides practical guidance about including people with disabilities, older adults, and caregivers in COVID-19 hygiene behavior change efforts. Still, it needs further testing to assess its appropriateness and effectiveness.

## Data availability statement

The original contributions presented in the study are included in the article/Supplementary material, further inquiries can be directed to the corresponding author.

## Author contributions

JW: conceptualization, methodology, data curation, supervision, project management, coordination, administration, funding acquisition, writing—review and editing, and visualization. JW and LW: investigation. JW, LW, and SF: writing—original draft preparation and manuscript review. All authors have read and approved the manuscript.

## Funding

The FCDO funds the review through PENDA (Programme for Evidence to Inform Disability Action) - a research consortium led by the International Center for Evidence in Disability at LSHTM (Grant Number PO8073). It was also supported by the Australian Government, Department of Foreign Affairs and Trade's Water for Women Fund (Grant Number WRA1).

## Conflict of interest

The authors declare that the research was conducted in the absence of any commercial or financial relationships that could be construed as a potential conflict of interest.

## Publisher's note

All claims expressed in this article are solely those of the authors and do not necessarily represent those of their affiliated organizations, or those of the publisher, the editors and the reviewers. Any product that may be evaluated in this article, or claim that may be made by its manufacturer, is not guaranteed or endorsed by the publisher.

## References

[1] World Health Organization World Bank. World Report on Disability. Geneva, Switzerland: WHO (2011). Available online at: https://www.who.int/teams/noncommunicable-diseases/sensory-functions-disability-and-rehabilitation/world-report-on-disability

[2] Office of the United Nations High Commissioner for Human Rights. Convention on the Rights of Persons with Disabilities. (2008). Available online at: https://www.ohchr.org/en/instruments-mechanisms/instruments/convention-rights-persons-disabilities#2 (accessed September 5, 2022).

[3] United Nations Department of Economic Social Affairs. World Population Ageing 2020 Highlights: Living Arrangements of Older Persons. New York, NY: United Nations, Population Division (2020). Available online at: https://www.un.org/development/desa/pd/sites/www.un.org.development.desa.pd/files/undesa_pd-2020_world_population_ageing_highlights.pdf

[4] World Health Organisation. Disability and Health. World Health Organisation (2021). Available online at: https://www.who.int/news-room/fact-sheets/detail/disability-and-health (accessed March 3, 2022).

[5] HelpAge International, Center for Community Development, Solutions,. Zimbabwe - August 2020, COVID-19 Rapid Needs Assessment of Older People. (2020). Available online at: https://reliefweb.int/report/zimbabwe/zimbabwe-covid-19-rapid-needs-assessment-older-people-august-2020 (accessed February 1, 2022).

[6] HelpAge International, Gravis,. Rajisthan, India - August 2020. COVID-19 Rapid Needs Assessment of Older People. (2020). Available online at: https://www.helpage.org/what-we-do/coronavirus-covid19/covid19-rapid-needs-assessment-rnas/ (accessed March 14, 2022).

[7] HelpAge International. Kigoma, Tanzania - August 2020. COVID-19 Rapid Needs Assessment of Older People. (2020). Available online at: https://www.helpage.org/what-we-do/coronavirus-covid19/covid19-rapid-needs-assessment-rnas/ (accessed March 14, 2022).

[8] HelpAge Cambodia. Cambodia - August 2020. COVID-19 Rapid Needs Assessment of Older People. (2020). Available online at: https://www.helpage.org/what-we-do/coronavirus-covid19/covid19-rapid-needs-assessment-rnas/ (accessed March 14, 2022).

[9] HelpAge International. Uganda, Adjumani Refugee Settlement - August 2020. COVID-19 Rapid Needs Assessment of Older People in Refugee Settlements. (2020). Available online at: https://www.helpage.org/what-we-do/coronavirus-covid19/covid19-rapid-needs-assessment-rnas/ (accessed March 14, 2022).

[10] Niu S Tian S Lou J Kang X Zhang L Lian H. Clinical characteristics of older patients infected with COVID-19: a descriptive study. Arch Gerontol Geriatr. (2020) 89:104058. 10.1016/j.archger.2020.104058 32339960 PMC7194515

[11] Williamson EJ Walker AJ Bhaskaran K Bacon S Bates C Morton CE. Factors associated with COVID-19-related death using OpenSAFELY. Nature. (2020) 584:430–6. 10.1038/s41586-020-2521-4 32640463 PMC7611074

[12] Kamalakannan S Bhattacharjya S Bogdanova Y Papadimitriou C Arango-Lasprilla JC Bentley J. Health risks and consequences of a COVID-19 infection for people with disabilities: scoping review and descriptive thematic analysis. Int J Environ Res Public Health. (2021) 18:4348. 10.3390/ijerph18084348 33923986 PMC8074171

[13] Glover C. Public Health England. Deaths of People Identified as Having Learning Disabilities with COVID-19 in England in the Spring of 2020. London: Public Health England – GOV.UK (2020). Available online at: https://assets.publishing.service.gov.uk/government/uploads/system/uploads/attachment_data/file/933612/COVID-19__learning_disabilities_mortality_report.pdf

[14] World Health Organization. Advice for the Public: Coronavirus Disease (COVID-19). World Health Organisation (2022). Available online at: https://www.who.int/emergencies/diseases/novel-coronavirus-2019/advice-for-public (accessed May 11, 2022).

[15] Global WASH Cluster (GWC). Global WASH Cluster - COVID 19 Response Guidance Note #02 - *Update 15 April 2020*. (2020). Available online at: https://drive.google.com/file/d/1uYAuqc-XTP6KEAFCpZR8lox12KhqhnhV/view (accessed October 13, 2022).

[16] World Health Organization UNICEF. Water, Sanitation, Hygiene, and Waste Management for SARS-CoV-2, the Virus That Causes COVID-19. (2020). Available online at: https://www.who.int/publications/i/item/WHO-2019-nCoV-IPC-WASH-2020.4 (accessed October 13, 2022).

[17] Meaney-Davis J, Lee, HNC,. The Impacts of COVID-19 on People with Disabilities (No. 35). London: Disability Inclusion Helpdesk (2020). Available online at: https://asksource.info/resources/impacts-covid-19-people-disabilities-a-rapid-review-disability-inclusion-helpdesk-query-no

[18] Kuper H Banks LM Bright T Davey C Shakespeare T. Disability-inclusive COVID-19 response: what it is, why it is important and what we can learn from the United Kingdom's response. Wellcome Open Res. (2020) 5:79. 10.12688/wellcomeopenres.15833.1 32500099 PMC7236579

[19] Mactaggart I Baker S Bambery L Iakavai J Kim MJ Morrison C. Water, women and disability: Using mixed-methods to support inclusive wash programme design in Vanuatu. Lancet Reg Health. (2021) 8:100109. 10.1016/j.lanwpc.2021.100109 34327430 PMC8315363

[20] Mactaggart I Schmidt WP Bostoen K Chunga J Danquah L Halder AK. Access to water and sanitation among people with disabilities: results from cross-sectional surveys in Bangladesh, Cameroon, India and Malawi. BMJ Open. (2018) 8:e020077. 10.1136/bmjopen-2017-020077 29866723 PMC5988144

[21] White S Kuper H Itimu-Phiri A Holm R Biran A. A qualitative study of barriers to accessing water, sanitation and hygiene for disabled people in Malawi. PLoS ONE. (2016) 11:e0155043. 10.1371/journal.pone.0155043 27171520 PMC4865162

[22] Banks LM White S Biran A Wilbur J Neupane S Neupane S. Are current approaches for measuring access to clean water and sanitation inclusive of people with disabilities? Comparison of individual- and household-level access between people with and without disabilities in the Tanahun district of Nepal. PLoS ONE. (2019) 14:e0223557. 10.1371/journal.pone.0223557 31603926 PMC6788693

[23] Sheppard P Polack S McGivern M. Mission millions: How older people with disabilities are excluded from humanitarian response. Help Age International, the International Centre for Evidence in Disability, the London School of Hygiene and Tropical Medicine. (2018). Available online at: https://www.lshtm.ac.uk/media/23446 (accessed January 5, 2022).

[24] Richard D Kiani S. Rapid Review of Disability Older Age Inclusion in Humanitarian WASH Interventions. (2019). Available online at: https://www.elrha.org/researchdatabase/rapid-review-of-disability-and-older-age-inclusion-in-wash/ (accessed March 6, 2022).

[25] Wilbur J Jones H Gosling L Groce N Challenger E. Undoing Inequity: inclusive water, sanitation and hygiene programmes that deliver for all in Uganda and Zambia. In: 336th WEDC International Conference. Nakuru, Kenya: WEDC (2013).

[26] Wilbur J Kayastha S Mahon T Torondel B Hameed S Sigdel A. Qualitative study exploring the barriers to menstrual hygiene management faced by adolescents and young people with a disability, and their carers in the Kavrepalanchok district, Nepal. BMC Public Health. (2021) 21:476. 10.1186/s12889-021-10439-y 33691653 PMC7944905

[27] HelpAge International Nsindagiza Organization. Rwanda - August 2020. COVID-19 Rapid Needs Assessment of Older People. (2020). Available online at: https://www.helpage.org/what-we-do/coronavirus-covid19/covid19-rapid-needs-assessment-rnas/ (accessed March 14, 2022).

[28] HelpAge International Oxfam. Iraq - August 2020. COVID-19 - Impact on Older People - Rapid Needs Assessment. (2020). Available online at: https://www.helpage.org/what-we-do/coronavirus-covid19/covid19-rapid-needs-assessment-rnas/ (accessed March 14, 2022).

[29] Wilbur J Torondel B Hameed S Mahon T Kuper H. Systematic review of menstrual hygiene management requirements, its barriers and strategies for disabled people. PLoS ONE. (2019) 14:e0210974. 10.1371/journal.pone.0210974 30726254 PMC6365059

[30] Wilbur J Morrison C Bambery L Tanguay J Baker S Sheppard P. “I'm scared to talk about it”: exploring experiences of incontinence for people with and without disabilities in Vanuatu, using mixed methods. Lancet Reg Health. (2021) 14:100237. 10.1016/j.lanwpc.2021.100237 34528002 PMC8355917

[31] Vaitheswaran S Lakshminarayanan M Ramanujam V Sargunan S Venkatesan S. Experiences and needs of caregivers of persons with dementia in India during the COVID-19 pandemic-A qualitative study. Am J Geriatr Psychiatry. (2020) 28:1185–94. 10.1016/j.jagp.2020.06.026 32736918 PMC7340037

[32] Pavlopoulou G Wood R Papadopoulos C. Impact of Covid-19 on the Experiences of Parents Family Carers of Autistic Children Young People in the UK. UC Institute of Education. (2020). Available online at: https://discovery.ucl.ac.uk/id/eprint/10101297 (accessed May 1, 2022).

[33] OHCHR. United Nations Principles for Older Persons. Adopted by General Assembly resolution 46/91 of 16 December 1991. (1991). Available online at: https://www.ohchr.org/en/professionalinterest/pages/olderpersons.aspx (accessed July 1, 2022).

[34] de Albuquerque C. Realising the Human Rights to Water Sanitation: a handbook by the UN Special Rapporteur Catarina de Albuquerque. (2014). Available online at: https://www.ohchr.org/en/special-procedures/sr-water-and-sanitation/handbook-realizing-human-rights-water-and-sanitation (accessed April 7, 2022).

[35] CBM International HelpAge International. Humanitarian Inclusion Standards for Older People and People with Disabilities. London, UK (2018).

[36] United Nations DoEaSA. Resolution adopted by the General Assembly on 28 July 2010; 64/292. The human right to water and sanitation. (2010). Available online at: https://digitallibrary.un.org/record/687002?ln=en (accessed November 1, 2022).

[37] Scherer N Mactaggart I Huggett C Pheng P. Rahman M-u, Wilbur J. Are the rights of people with disabilities included in international guidance on WASH during the COVID-19 pandemic? Content analysis using EquiFrame. BMJ Open. (2021) 11:e046112. 10.1136/bmjopen-2020-046112 34257092 PMC8282413

[38] Wilbur J. COVID-19 Inclusive WASH Checklist. London, UK: HygieneHub. (2020).

[39] Scherer N Mactaggart I Huggett C Pheng P Rahman MU Biran A. The inclusion of rights of people with disabilities and women and girls in water, sanitation, and hygiene policy documents and programs of Bangladesh and Cambodia: content analysis using equiframe. Int J Environ Res Public Health. (2021) 18. 10.3390/ijerph18105087 34064939 PMC8151976

[40] Rapporteur US. Frequently Asked Questions: The Special Rapporteur on the Human Right to Safe Drinking Water and Sanitation. In: Ohchr, editor. Geneva (2021).

[41] de Albuquerque C,. Realising the Human Rights to Water Sanitation: A Handbook by the UN Special Rapporteur: Principles. (2014). Available online at: https://www.ohchr.org/en/special-procedures/sr-water-and-sanitation/handbook-realizing-human-rights-water-and-sanitation (accessed May 1, 2022).

[42] Australian Aid Water for Women, CBM,. Disability Inclusion COVID-19: Guidance for WASH Delivery. (2020). Available online at: https://www.cbm.org.au/resource/disability-inclusion-and-covid-19-guidance-for-wash-delivery (accessed May 17, 2022).

[43] WaterAid. Key Tool: Equity, Non-discrimination and Inclusion in WASH Checklist. WASH Matters. (2018). Available online at: https://washmatters.wateraid.org/publications/equality-non-discrimination-and-inclusion-toolkit (accessed May 15, 2022).

[44] Wilbur J. Summary Report on Considering Disability Ageing in COVID-19 Hygiene Promotion Programmes HygieneHub. (2020). Available online at: https://resources.hygienehub.info/en/articles/4097594-summary-report-on-considering-disability-and-ageing-in-covid-19-hygiene-promotion-programmes (accessed August 1, 2022).

[45] Statistics. WGoD. The Washington Group Extended Set on Functioning (WG-ES). (2020). Available online at: https://www.washingtongroup-disability.com/fileadmin/uploads/wg/Documents/Questions/Washington_Group_Questionnaire__2_-_WG_Extended_Set_on_Functioning.pdf (accessed February 22, 2022).

[46] Department for International Development (DFID). DFID's Strategy for Disability Inclusive Development 2018-23. London, England: DFID (2018).

[47] PENDA (Programme for Evidence to Inform Disability, Action), LSHTM,. Hygiene and Behaviour Change: Inclusion of People with Disabilities and Older People in COVID-19 Response (London). (nd). Available online at: https://www.lshtm.ac.uk/research/centres-projects-groups/penda#research (accessed January 31, 2022).

[48] Wilbur J Scherer N Mactaggart I Shrestha G Mahon T Torondel B. Are Nepal's water, sanitation and hygiene and menstrual hygiene policies and supporting documents inclusive of disability? A policy analysis. Int J Equ Health. (2021) 20. 10.1186/s12939-021-01463-w 34238285 PMC8268379

[49] Ansari Z White S. Managing incontinence in low-and middle income-countries: A qualitative case study from Pakistan. PloS one. (2022) 17:e0271617. 35839232 10.1371/journal.pone.0271617 PMC9286225

[50] Wilbur J Pheng P Has R Banks L Huggett C Scherer N. A qualitative cross-sectional study exploring the implementation of disability-inclusive WASH policy commitments in Svay Reing and Kampong Chhnang Provinces, Cambodia. Front Water. (2022) 4. 10.3389/frwa.2022.963405

[51] Wilbur J Morrison C Iakavai J Shem J Poilapa R Bambery L. “The weather is not good”: exploring the menstrual health experiences of menstruators with and without disabilities in Vanuatu. Lancet Reg Health. (2021) 18. 10.1016/j.lanwpc.2021.100325 35024657 PMC8661049

[52] Pryor W Marella M Robinson A. Gap Analysis: the inclusion of People with Disability and Older People in Humanitarian Response. Part 2 Beyond the Evidence: Implications for Innovation And Practice. London, UK: Elrha. (2020).

[53] Sphere. The Sphere Handbook. Humanitarian Charter and Minimum Standards in Humanitarian Response. (2018). Available online at: https://spherestandards.org/handbook-2018/ (accessed September 22, 2022).

[54] United Nations. Resolution adopted by the General Assembly on 3 June 2015. 69/283. Sendai Framework for Disaster Risk Reduction 2015–2030. Geneva, Switzerland: United Nations. (2015).

[55] CHS Alliance, Group, URD, Sphere, Project. (2014). Core Humanitarian Standard on Quality and Accountability. Available online at: https://corehumanitarianstandard.org/files/files/Core%20Humanitarian%20Standard%20-%20English.pdf (accessed October 24, 2022).

[56] Aunger R Curtis V. Behaviour centred design: towards an applied science of behaviour change. Health Psychol Rev. (2016) 10:425–46. 10.1080/17437199.2016.1219673 27535821 PMC5214166

[57] White S Schmidt W Sahanggamu D Fatmaningrum D van Liere M Curtis V. Can gossip change nutrition behaviour? Results of a mass media and community-based intervention trial in East Java, Indonesia. Trop Med Int Health. (2016) 21:348–64. 10.1111/tmi.12660 26701153

[58] Farrelly MC Healton CG Davis KC Messeri P Hersey JC Haviland ML. Getting to the truth: evaluating national tobacco countermarketing campaigns. Am J Public Health. (2002) 92:901–7. 10.2105/AJPH.92.6.901 12036775 PMC1447480

[59] Hornik R Yanovitzky I. Using theory to design evaluations of communication campaigns: the case of the national youth anti-drug media campaign. Commun Theory. (2003) 13:204–24. 10.1111/j.1468-2885.2003.tb00289.x 25525317 PMC4267481

[60] Pechmann C Reibling ET. Antismoking advertisements for youths: an independent evaluation of health, counter-industry, and industry approaches. Am J Public Health. (2006) 96:906–13. 10.2105/AJPH.2004.057273 16571709 PMC1470598

